# Improvements in survival of the uncemented Nottingham Total Shoulder prosthesis: a prospective comparative study

**DOI:** 10.1186/1471-2474-8-76

**Published:** 2007-08-04

**Authors:** Nahum Rosenberg, Lars Neumann, Amit Modi, Istvan J Mersich, Angus W Wallace

**Affiliations:** 1 The Nottingham Shoulder and Elbow Unit, City Hospital, Nottingham, UK

## Abstract

**Background:**

The uncemented Nottingham Total Shoulder Replacement prosthesis system (Nottingham TSR) was developed from the previous BioModular^® ^shoulder prosthesis taking into consideration the causes of the initial implant's failure.

We investigated the impact of changes in the design of Nottingham TSR prosthesis on its survivorship rate.

**Methods:**

Survivorship analyses of three types of uncemented total shoulder arthroplasty prostheses (BioModular^®^, initial Nottingham TSR and current Nottingham TSR systems with 11, 8 and 4 year survivorship data respectively) were compared. All these prostheses were implanted for the treatment of disabling pain in the shoulder due to primary and secondary osteoarthritis or rheumatoid arthritis. Each type of the prosthesis studied was implanted in consecutive group of patients – 90 patients with BioModular^® ^system, 103 with the initial Nottingham TSR and 34 patients with the current Nottingham TSR system.

The comparison of the annual cumulative survivorship values in the compatible time range between the three groups was done according to the paired *t *test.

**Results:**

The 8-year and 11-year survivorship rates for the initially used modified BioModular^® ^uncemented prosthesis were relatively low (75.6% and 71.7% respectively) comparing to the reported survivorship of the conventional cemented implants. The 8-year survivorship for the uncemented Nottingham TSR prosthesis was significantly higher (81.8%), but still not in the desired range of above 90%, that is found in other cemented designs. Glenoid component loosening was the main factor of prosthesis failure in both prostheses and mainly occurred in the first 4 postoperative years. The 4-year survivorship of the currently re-designed Nottingham TSR prosthesis, with hydroxylapatite coating of the glenoid baseplate, was significantly higher, 93.1% as compared to 85.1% of the previous Nottingham TSR.

**Conclusion:**

The initial Nottingham shoulder prosthesis showed significantly higher survivorship than the BioModular^® ^uncemented prosthesis, but lower than expected. Subsequently re-designed Nottingham TSR system presented a high short term survivorship rate that encourages its ongoing use

## Background

Inflammatory or degenerative processes of glenohumeral joint lead to pain and restriction of movements of the shoulder. Prosthetic replacement of the glenohumeral joint has gained in popularity because of its efficacy in relieving pain. The pioneering successful prostheses for total shoulder arthroplasty have been based on an unconstrained design, i.e. a metal spherical head component fixed to a metal intramedullary stem articulating with a high-density polyethylene socket (Table 1). These components are stabilized in the adjacent bone using polymethylmethacrylate bone cement [1]. The important cause for failure of the cemented prostheses was related to the glenoid component, with a 0.01–6% rate of loosening [2-4].

**Table 1:** Long term survivorship data on cemented and the outcome of a large series of a cementless total shoulder replacement prostheses

**Reference**	**Type of prosthesis**	**No. of Patients**	**Survivorship**	**End point criteria**	**Glenoid failure rate**	**Overall failure rate**
Tarchia, Cofield & Settergren [5]	Neer I & II cemented	**113** [31 = OA, 36 = RA 12 = 2ary OA]	10years = 93% 15years = 87%	Revision – severe pain, abd < 90°, ext rot < 20°	7/113	14/113
Brenner, Perlic, Clayton & Dennis [8]	Neer II & Gristina cemented	**51** [37 = OA 14 = RA]	11years = 75%	Severe pain, radiographic evidence of component loosening	3/51	6/51
Cofield [6]	Cofield cementless	**180** [110 = OA 28 =RA 30 = 2ary OA 12 = revisions]	Not calculated	Revision	5/180	12/180
Pfahler et al [4]	Aequalis cemented	**705** [418 = OA 107 = RA 180 = 2ary OA]	Not calculated	Revision	9/705	43/705

OA = osteoarthritis, RA = rheumatoid arthritis

The long term survivorship of the prosthesis developed by C. Neer for the cemented total shoulder arthroplasty (TSA) is almost the single one with well documented outcomes [5] with 87% fifteen year survivorship rate for Neer I & II cemented shoulder prostheses. This implant has become the gold standard, against which all the successive prosthetic designs are compared.

Further developments of TSA implants have been aimed at enhancing longevity by addressing the following three most critical issues: (1) Improving the incorporation of the glenoid component using a more "biological" type of fixation in order to reduce the rate of mechanical loosening; (2) Designing a better glenoid component to achieve the lowest possible rate of wear; (3) Finding the best method for the fixation of the humeral component while allowing good preservation of the humeral bone stock, taking into consideration need for possible future revision surgery.

These goals can potentially be achieved using an uncemented design, with press fit and/or tissue in-growth porous coating of the metal, at its bone interface. There is evidence that the porous coating at the proximal part of the stem is superior to the press fit design [6], possibly because of lesser stress shielding of the proximal humerus and subsequently less bone resorption, and preservation of the proximal humeral bone stock. Biological fixation of the glenoid component currently requires the use of a metal backing or metal base-plate that serves as the "bone in-growth" surface and results in a more even distribution of the compression forces on the bone. On this baseplate a high-density polyethylene insert is mounted, either molded onto the metal or fixed using some form of fastening mechanism at the time of surgery. This bearing should be at least 3 mm thick (at its thinnest part) to reduce polyethylene wear [2,7].

Currently there are very few long-term peer reviewed large series survivorship data on cemented TSAs [5,8] and no survivorship data on cementless designs (Table 1). Survivorship studies with commonly acceptable clinical outcome criteria are important, but currently there is no uniform agreement on these types of criteria. Most authors consider revision of the prosthesis an end point in its survivorship [9]. Furthermore published reports on single prosthetic designs so far have only provided short-term postoperative follow-up data or a small number of patients. All these factors result in wide confidence intervals in survivorship tables and lead to difficulty in drawing a meaningful interpretation of the results [9].

In spite of these problems we can reach some tentative conclusions from TSA outcome by different authors (Table 1). The best long-term outcome is that reported for the Neer II cemented prosthesis, with a 93% ten years survivorship. Short-term glenoid failure, requiring implant removal, reaches the rate of around 6% for cemented designs and 3% for cementless designs. Glenoid failure is the cause of between 20% – 50% of all failed TSAs, cemented or cementless. Survivorship of cemented TSA is highest in patients with rheumatoid arthritis. We are nor aware of survivorship data available for rheumatoid patients with cementless designs. It is logical to conclude that any cementless prosthetic design should possess at least the best survivorship characteristics of the cemented prostheses in order to be considered as an alternative.

**Figure 1:**
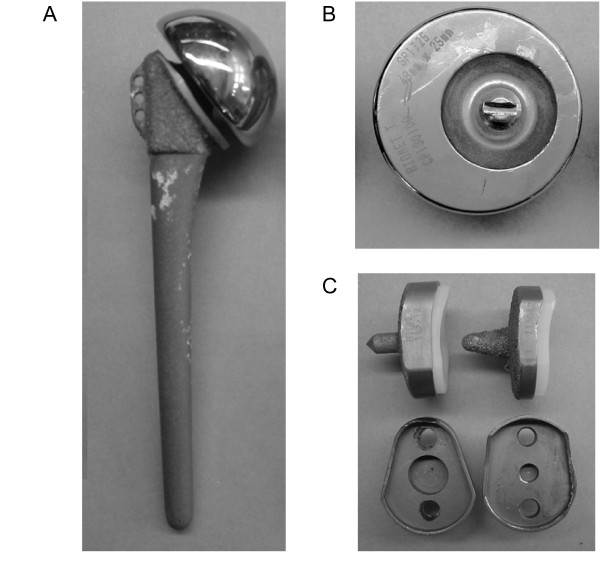
The BioModular^® ^total shoulder arthroplasty prosthetic design is shown. (A) A titanium BioModular^® ^stem. (B) An offset "Wallace" head. (C) The glenoid trays: on the right – the low-profile version, top row – view from the side, bottom row – view into the tray, showing the glenoid liner capture mechanisms.

**Figure 2:**
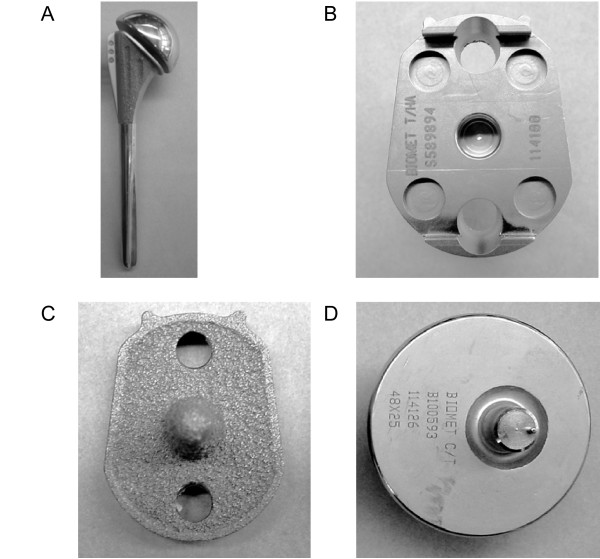
The Nottingham TSR total shoulder arthroplasty prosthetic design is shown. (A) A chrome cobalt stem. (B) A glenoid tray showing the capture mechanism for the polyethylene liner. (C) A glenoid tray seen from the back, showing the areas for bone in-growth. (D) An off-set head with a morse taper assembling interface.

**Figure 3:**
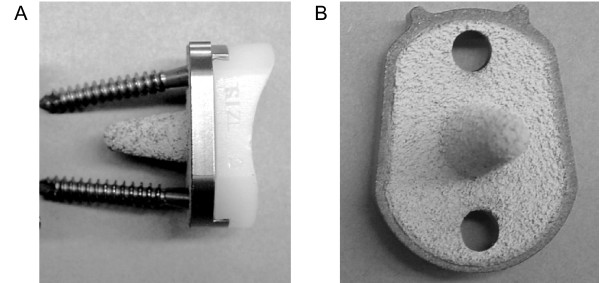
The Nottingham TSR total shoulder arthroplasty prosthetic design with glenoid base-plate coated with hydroxyapatite. (A) A lateral view of the base-plate with fixation screws and with mounted polyethylene liner. (B) A view on the bone interface side of the base-plate covered by hydroxyapatite.

In order to achieve the goal of the desired survivorship rates of cementless TSA a chain of modification of the initial BioModular^® ^prosthesis was employed with eventual evolvement of the current Nottingham TSR design. The main characteristics of this prosthesis are: (1) The use of an indexable offset modular head, to improve the anatomical configuration of the implant and the optimal soft tissue balancing [10,11]. (2) A porous proximal stem in order to eliminate the stress shielding effect. (3) Conformed radii of humeral and glenoid components in order to reduce point loading and point wear of the polyethylene glenoid liner [12]. (4) Hydroxyapatite lining of the glenoid baseplate-bone interface in order to provide a "biologic" milieu to improve an osseo-integration [13]. (5) An improved capture mechanism for holding the polyethylene bearing onto the base-plate in order to reduce the liner disengagement rate. Following these design changes we hypothesize that the TSA with cementless implantation will present improved survivorship rates. Therefore in order to estimate the improvement in a performance of the prostheses design we have compared survivorship data of the BioModular^® ^prosthesis – with or without the "Wallace" prototype offset head (Fig 1) – with the survivorship of the initial design of the Nottingham TSR (Fig 2) and the latest design of the Nottingham TSR with the glenoid component base-plate covered by hydroxyapatite (Fig 3). We show that Nottingham TSR uncemented prosthesis has better short and midterm survivorship than the BioModular^® ^uncemented design.

## Methods

Survivorship analyses of three types of uncemented TSA prostheses, implanted for the treatment of disabling pain in the shoulder due to primary and secondary osteoarthritis or rheumatoid arthritis, were compared. Each type of the prosthesis studied was implanted in consecutive group of patients, i.e. Group 1: the BioModular^® ^TSA prosthesis, Group 2: The initial Nottingham TSR prosthesis and Group 3: The most recently redesigned Nottingham TSR prosthesis. The indication for surgery and criteria for inclusion in the study were pain in the shoulder with functional disability combined with radiographic evidence of an advanced destruction of the humeral and glenoid articular surfaces. Patients compatible with these criteria but who were medically unfit for surgery, due to advanced systemic disease, were not offered the procedure and were therefore excluded from the study.

Group 1 was comprised of 90 patients who were operated between 1989–1994 (15 men and 75 women, mean age 61 years, range 19 – 92 years). These patients had the uncemented BioModular^® ^Total Shoulder Prostheses implanted, either with the standard non-offset head or the prototype ("Wallace") offset humeral head. The mean follow up period in this group was 8.8 years.

Group 2 patients were treated with the initial Nottingham TSR cementless prosthesis where the glenoid component had no hydroxyapatite backing. This group included 103 patients – 12 men and 91 women, average age of 58 years (range: 20–84 years). This design has been used between 1994 and 1997 and the patients had a mean follow up period of 6.4 years.

Group 3 comprised of 34 patients, who had a hydroxyapatite coated glenoid component base plate implanted, as part of their most recent Nottingham TSR. In this group there were two men and 32 women, with a mean age of 64 years (range 31–89 years). These patients were operated in 1998–99 and had a mean follow up of 3.2 years.

The characteristics of the different groups of patients are given in the Table 2. All these patients were included in the survivorship analyses.

**Table 2:** Characteristics of the study groups

**Study Group**	**Age (years)**	**Male/Female**	**Mean follow up (years)**	**OA**	**RA**	**2**^nd^**ary OA**	**Total Number**
**Group 1**	Mean: 61 Range: 19–92	15/75	8.8	48 (1 with RC tear)	31 (2 with RC tear)	11	90
**Group 2**	Mean: 58 Range: 20–84	12/91	6.4	47	36 (2 with RC tear)	20	103
**Group 3**	Mean: 64 Range: 31–89	2/32	3.2	19	12 (1 with RC tear)	3	34

OA = osteoarthritis, RA = rheumatoid arthritis, RC – rotator cuff muscle

For the comparison of the three TSA prostheses' clinical outcome we used a survivorship analysis according to the method described by Murray et al [14] which is based on Rothman's formula for the confidence limits determination. The criterion for failure in this series was revision surgery requiring removal or exchange of either part of or a whole prosthesis. The indications for these re-operations were: (1) An increased level of pain during follow-up, that appeared to be related to the implant, with restriction of external rotation to under 20° and abduction to under 60° and/or newly developed radiolucency around the glenoid peg or complete peri-prosthetic radiolucency at the metal-bone interface, more then 2 mm in width, around either the humeral or glenoid components [7]. The radiographic evaluation was done by the surgical team and by the radiologist; (2) Deep wound infection; (3) Migration of any of the prosthetic components.

For the purpose of postoperative follow up and identification of the possible failure of the implants the patients were monitored annually. This review evaluation included estimation of the level of pain using a Visual Analog Scale, a clinical examination of the range of movements of the shoulder and radiographic evaluation of the shoulder with an anterior – posterior view and an axillary view to allow assessment of the alignment and position of the components, the presence of any change in position over time and measurement of any radiolucency at the prosthesis-bone interface.

Information on patients who died during the follow up period, which is included in the survivorship analysis, was obtained either from the Registrar for Births, Marriages and Deaths or from the hospitals' registration systems and through direct contact with General Practitioners or relatives.

The comparison of the annual cumulative survivorship values (quantitative type of variables) in the compatible time range between the three groups was done according to the paired *t *test and the p < 0.05 was considered significant.

The TSA operations were performed through a proximally extended deltopectoral approach with a longitudinal clavicle osteotomy and a lesser tuberosity osteotomy [15]. This approach was used to facilitate glenoid exposure and to ensure stable deltoid and subscapularis muscle reattachment, that allows early postoperative shoulder mobilization [16]. The authors prefer this surgical approach also because it protects the deltoid muscle during retraction. After humeral head resection, humeral stem alignment was established using the anatomical neck as a guide to prosthesis placement, preserving the rotator cuff. Humeral medullary canal and glenoid surface preparation were carried out using specially designed reamers. With the standard BioModular^® ^stems either a standard or an offset prototype modular head was used. The head geometry, apart from the off-set feature, was identical in the two types of prostetic heads used with the BioModular^® ^implant. In the Nottingham TSR system humeral stems in four sizes, offset modular heads in five sizes and glenoid bearings in three thicknesses were available for optimal component fitting and soft tissue balancing. In six patients (three in the Group 1, two in the Group 2 and one patient in the Group 3 – Table 2) tears in rotator cuff muscles were identified and firmly repaired. Five of six patients with rotator cuff muscles tears suffered from the rheumatoid arthritis.

During the first three months postoperatively every patient underwent intensive physiotherapy following a standard programme aimed at improving strength and range of movements. Since the surgical procedure was carried out through a deltopectoral approach with a lesser tuberosity osteotomy and its strong reattachment, an immediate postoperative rehabilitation was possible with very few limitations. Usually a work on external rotation and elevation was commenced from the first postoperative day with isometrics, passive and active elevation and external rotation up to the level of movement achieved during the surgery. Patients used the broad arm sling intermittently only during the first 2–3 postoperative days. The exercise programme was increased as the patients gain confidence and pain relief aiming to achieve a maximal possible active and passive range of movements.

## Results

**Figure 4:**
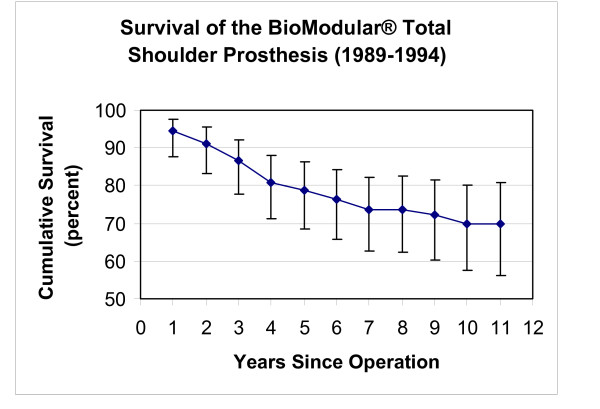
This graph shows the survival results of BioModular^® ^total shoulder arthroplasty (vertical bars represent 95% confidence intervals).

**Figure 5:**
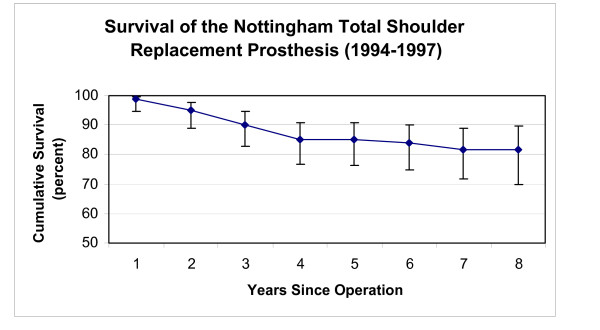
This graph shows the survival rate of the initial design for the Nottingham TSR (vertical bars represent 95% confidence intervals).

The eight year survivorship of the initial Nottingham TSR design (Group 2) was higher (p < 0.001) than observed in Group 1 patients with implanted BioModular^® ^prostheses (Fig 4,5). The eight-year cumulative survivorship in the Group 2 was 81.8% and remained constant from the sixth postoperative year (Fig 5). The eight – and ten- year cumulative survival rates of Group 1 (BioModular^® ^prosthesis) were 75.6% and 71.7% (Fig 4). The main causes of failure of the BioModular^® ^prosthesis were related to the glenoid component, i.e. aseptic glenoid component loosening in 13 patients and uncoupling of the polyethylene bearing liner in 4 patients, overall 71% of failed cases (Table 3). Seventy one percent of the failed cases occurred during the first four postoperative years (Table 3) showing four year cumulative survivorship rate of 80.9% (Fig 4). The main drop in survivorship of the initial Nottingham TSR occurred also during the initial four postoperative years and was mainly due to glenoid component failure (11 of the 17 failed cases, Table 4). About half of the failures in Group 2 during the eight years of the survivorship analysis were due to aseptic loosening of the glenoid base-plate in eight patients, six of these patients were treated for primary or secondary osteoarthritis (Table 5).

**Table 3:** Time distribution of the occurrence of prostheses failure in the Group 1 (patients operated in 1989 – 94 with BioModular^® ^uncemented TSA) according to the mode of failure

**Year Post Op**	**Glenoid Loosening**	**Bearing failure**	**Infection**	**Dislocation**
1	**3**	**1**	**0**	**1**
2	**1**	**1**	**0**	**1**
3	**0**	**0**	**1**	**3**
4	**3**	**1**	**1**	**0**
5	1	0	0	0
6	2	0	0	0
7	2	0	0	0
8	0	0	0	0
9	1	0	0	0
10	0	1	0	0
11	0	0	0	0
Total	13	4	2	5

**Table 4:** Time distribution of the occurrence of prostheses failure in the Group 2 (patients operated in 1994 – 97 with the initial Nottingham TSR prosthesis) according to the mode of failure

**Year Post Op**	**Glenoid Loosening**	**Bearing failure**	**Infection**	**Dislocation**
1	**0**	**1**	**0**	**0**
2	**0**	**3**	**0**	**1**
3	**2**	**1**	**0**	**2**
4	**4**	**0**	**0**	**1**
5	0	0	0	0
6	1	0	0	0
7	1	0	0	0
8	0	0	0	0
Total	8	5	0	4

**Table 5:** Failed uncemented Nottingham TSR prostheses in Group 2 (patients operated in 1994 – 97) according to their mode of failure

**Component**	**Mode of Failure**		
	**Loosening**	**Infection**	**Malposition: bearing failure/head dislocation, stem malposition**

**Glenoid**	**8 **(OA = 4, RA = 2, 2^nd^ary OA = 2)	**0**	**7 **[OA = 4, 2^nd^ary OA = 3]
**Humeral**	**0**	**0**	**2 **[2^nd^ary OA = 2]

OA = osteoarthritis, RA = rheumatoid arthritis

**Figure 6:**
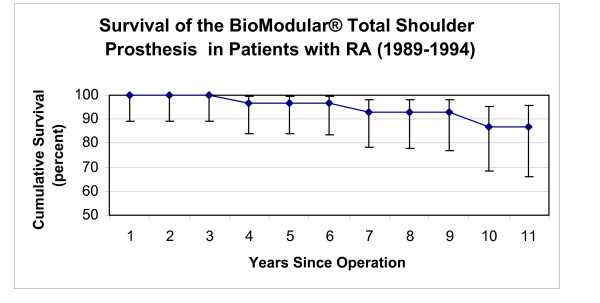
This graph shows the survival rates of the BioModular^® ^total shoulder arthroplasty in patients with RA (vertical bars represent 95% confidence intervals).

**Figure 7:**
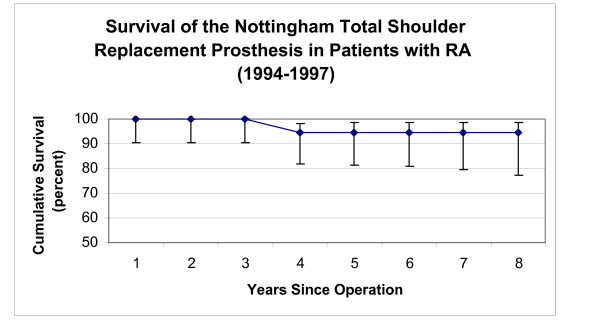
This graph shows the survival rate of the initial design of the Nottingham TSR in patients with RA (vertical bars represent 95% confidence intervals).

Survivorship in patients with rheumatoid arthritis (RA) in both groups 1 and 2 was higher (p < 0.001) than in patients with osteoarthritis. The patients with RA who had the BioModular^® ^prosthesis implanted, presented a 96.8% and 93.1% cumulative five and eight year survivorships respectively (Fig 6) and the RA patients with the initial Nottingham TSR had presented a constant 94.4% cumulative survivorships from the fourth to eights year postoperatively (Fig 7).

**Figure 8:**
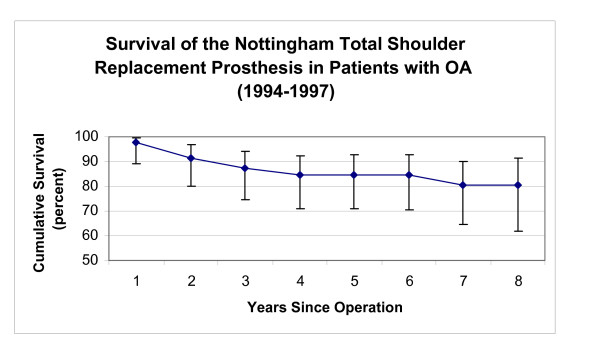
This graph shows the survival rate of the initial design for the Nottingham TSR in patients with OA (vertical bars represent 95% confidence intervals).

**Figure 9:**
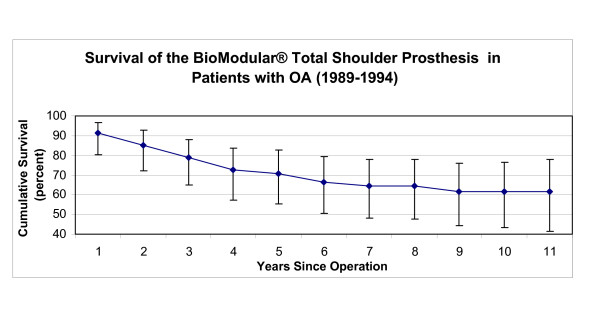
This graph shows the survival results of the BioModular^® ^total shoulder arthroplasty in patients with OA (vertical bars represent 95% confidence intervals).

The initial Nottingham TSR prostheses showed higher eight year survivorship than BioModular^® ^prostheses in patients with OA (p < 0.01). Survivorship of the BioModular^® ^prosthesis in patients with primary osteoarthritis was 70.9% and 64.3% at five and eight years respectively and for the initial Nottingham TSR these values were 84.5% and 80.4% (Fig 8,9).

**Figure 10:**
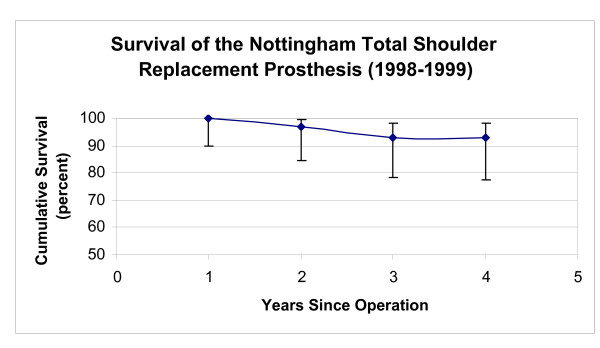
This graph shows the survival of the current Nottingham TSR with a hydroxyapatite coated glenoid baseplate (vertical bars represent 95% confidence intervals).

The observed four-year survivorship of the patients in Group 3 was also significantly higher than the survivorship rates of the BioModular^® ^prosthesis observed after the first four years after implantation (p = 0.02), with a 93.1% cumulative four year survivorship of the re-designed Nottingham TSR (Fig 10) comparing to the 80.9% of cumulative four year survivorship of the BioModular^® ^prosthesis (Fig 4). Among the Group 3 patients only two prostheses failed, both in patients with osteoarthritis (Table 6). One failure was due to glenoid bearing disassembly, two years after the operation, and the other due to glenohumeral dislocation three years after the operation (Table 7).

**Table 6:** Failed uncemented Nottingham TSR prostheses in the Group 3 (patients operated in 1998 -99) according to the mode of failure

**Component**	**Mode of Failure**		
	**Loosening**	**Infection**	**Malposition: bearing failure/head dislocation**

**Glenoid**	**0**	**0**	**2 **(OA = 2)
**Humeral**	**0**	**0**	**0**

OA = osteoarthritis

**Table 7:** Time distribution of the occurrence of prostheses failures in the Group 3 (patients operated in 1998 – 99 with the current Nottingham TSR prosthesis) according to the mode of failure

**Year Post Op**	**Glenoid Loosening**	**Bearing failure**	**Infection**	**Dislocation**
**1**	0	0	0	0
**2**	0	1	0	0
**3**	0	0	0	1
**4**	0	0	0	0
**Total**	0	1	0	1

## Discussion

The evolving cementless TSA prostheses should present longevity that is comparable with or better than conventionally used cemented implants. We evaluated the short and midterm survivorship of the Nottingham TSR cementless prosthesis, with comparison to the survivorship of the BioModular^® ^TSA prosthesis from which the Nottingham TSR evolved, in order to estimate the ability of these implants to achieve the desirable survivorship rates. The transitions from one design to another occurred following recognition of causes of implant failure. The use of the offset head on the standard BioModular^® ^stem design did not prevent a considerably high loosening rate. The subsequent change to a conforming design with an identical radii of curvature of the humeral head component and glenoid component and to a different glenoid component with a conical peg, allowing press-fit implantation, led to significant improvement in the middle term survivorship among the patients who had an implant of the initial Nottingham TSR design. We are aware of suggestions by other authors, that full conformity between the components may lead to an enhanced stress on the globoid rim due to loss of the humeral head translation possible in the normal shoulder joint, and as a consequence a higher risk for prosthetic loosening. [12,17]. Since this hypothesis has been raised following cadaveric studies and in vivo radiographic evaluation and has been never confirmed in clinical trials and since the exact degree of optimal mismatch of the glenoid and the humeral head radii is not known, the authors preferred the conforming design. This choice of conformity is based on the hypothesis that mismatch of the glenoid and humeral component curvature can lead to a considerable rate of polyethylene wear due to uneven force distribution between the components and point loading and point wear of the polyethylene [12].

The midterm survivorship in the Group 2 patients shows that the original Nottingham TSR had less favorable results when compared with the existing published survivorship studies on the Neer I & II cemented implants [5], with the outcome studies of the Cofield uncemented prostheses [6] and with the "Aequalis" cemented prosthesis [4], but is comparable with the study on the Neer II and Gristina prostheses [8]. The comparison with the latter study is more realistic because the groups of patients are more comparable to our patients, i.e.both series consist mainly of patients with primary osteoarthritis, smaller groups of patients with rheumatoid arthritis and some patients with secondary osteoarthritis, and they have similar indications for failure recognition. Although the midterm survivorship of the initial Nottingham TSR design showed a significant improvement over the survivorship rate of the BioModular^® ^prosthesis, especially in the patients with osteoarthritis, this improvement did not reach the desired values of survivorship above 90% that has been reported for the cemented Neer I&II prostheses. We note that the overall lower than desired midterm survivorship rates in the Group 2 patients are due to less favorable performance of the Nottingham TSR prosthesis in patients with primary osteoarthritis, who had an eight year survivorship rate of only 80.4%. The interesting finding is the evidence of a significantly higher (and in the desirable range of above 90%) survivorship in patients with rheumatoid arthritis compared to the patients with osteoarthritis in both Group 1 and Group 2. The difference in the survivorship between patients with osteoarthritis and rheumatoid arthritis is consistent with observations in other reports on other prosthetic shoulder arthroplasty systems [8] and can be explained by the lower level of demand placed on a shoulder prostheses in a rheumatoid patient.

In spite of the improved survivorship in the initial design of the Nottingham TSR it still showed an unsatisfactory short term loosening rate. It should be realized that the two most serious complications of any prosthetic surgery, e.g. deep wound infection and periprosthetic fracture, did not occur among any of the patients treated by the initial design of the Nottingham TSR. This can probably be attributed to the use of an appropriate surgical technique and instrumentation for the prosthesis implantation. The glenohumeral dislocation rate appeared to be similarly low in Group 1, five of 90 patients, and Group 2, four of 103 patients, (Tables 3,4). This finding shows that the tissue balancing technique for the Nottingham TSR prosthesis implantation, i.e. systematic intraoperative evaluation of an adequate free subacromial space, flush and stable alignment of the glenoid and humeral components with correct offset of the humeral head, adequate anterior – posterior laxity and ability to reattach the lesser tuberosity with the arm in external rotation without loosing of the desired glenohumeral reduction, was effective.

The main mode of failure of the prostheses in Group 2 patients was aseptic loosening of the glenoid component predominantly occurring in the first four postoperative years and the second most important cause of failure cause bearing disassembly during the same postoperative period (Table 4). After having identified the two main causes of failure for the intermediate design of the Nottingham TSR prosthesis, steps were taken to change of the design of the metal base-plate of the glenoid component. To achieve an optimal bone osseointegration coating of the implant with hydroxyapatite was introduced. In order to eliminate glenoid bearing disassembly improvement of the capture mechanism for the glenoid bearing was implimented. When looking at the data for Group 3, the addition of hydroxyapatite to the porous coating of the glenoid base-plate has eliminated the original 3.9–5.6% rate of aseptic glenoid loosening from the second to the fourth postoperative years (Tables 3, 4, 7). Additionally by improving the capture mechanism of the glenoid bearing its disassembly has now became rare (Tables 3, 4, 7). Following these changes in design the four-year survivorship of the Nottingham TSR prostheses in the Group 3 patients showed a satisfactory 93.1 % rate, which is significantly higher than the four-year survivorship rates of the BioModular^® ^system. Since in the Groups 1 and 2 the deterioration in the survivorships occurred predominantly in the first four postoperative years, the present high four-year survivorship rate of the newly designed prosthesis might indicate on a sustained long-term improvement of the prosthesis survivorship.

Since Group 3 comprised of only 34 patients we have not subdivided and compared the subgroups according to the underlying pathology as we did in Groups 1 and 2. Furthermore, because the number of patients in Group 3 is not sufficient for the adequate power of the statistical comparison with the survivorship in Group 2 patients we can only suggest that the further improvement of the present Nottingham TSR system survivorship is likely to be seen. Future long-term survivorship studies will verify this point more precisely. However, with regard to its initial design (Group 2), there is a clear evidence that the cementless Nottingham TSR system led to a significant improvement in its midterm survivorship comparing to its predecessor, the BioModular^® ^prosthesis.

## Conclusion

We have shown that the discussed cementless TSA prosthesis design (Nottingham TSR), following a chain of modifications according to recognition of previous causes of failure, have reached short term survivorship rates that are comparable to the conventional cemented designs. Therefore, in the light of potential long term "biological" advantages of uncemented implants, these results are encouraging for the ongoing use and development of this type of prosthesis.

## Abbreviations

TSA – total shoulder arthroplasty

TSR – total shoulder replacemen

## Competing interests

The authors has received research funding from Biomet Merck Ltd.

## Authors' contributions

NR – processed the data and wrote the paper.

LN – collected and analyzed the data.

AM – collected the data.

IJM – collected the data.

WAW – collected and analyzed the data.

## Pre-publication history

The pre-publication history for this paper can be accessed here:


